# Evolution of multidisciplinary obesity treatments: past, present, and future role of nutrition

**DOI:** 10.1002/oby.24340

**Published:** 2025-08-20

**Authors:** Steven B. Heymsfield, Philip J. Atherton, Sandra Christensen, Colleen Tewksbury, Amanda Velazquez, Jens Walter, Ellen E. Blaak

**Affiliations:** ^1^ Pennington Biomedical Research Center Louisiana State University System Baton Rouge Louisiana USA; ^2^ Royal Derby Hospital Derby UK; ^3^ Integrative Medical Weight Management Seattle Washington USA; ^4^ School of Nursing University of Pennsylvania Philadelphia Pennsylvania USA; ^5^ Cedars‐Sinai Los Angeles California USA; ^6^ University College Cork Cork Ireland; ^7^ Maastricht University Medical Center+ Maastricht Netherlands

## Abstract

Obesity is a complex chronic disease requiring lifelong comprehensive treatment. In addition to lifestyle counseling that improves nutrition and physical activity, a promising new generation of obesity medications has been added to bariatric procedures as therapeutic options to achieve weight reduction and improve health outcomes. With the promise of effective and safe treatments comes the need to emphasize maximal reduction of body fat and minimal loss of vital body components, including skeletal muscle and bone. Nutrition is a critical aspect of obesity care and is leveraged to support preservation of lean tissues, such as skeletal muscle, through adequate, daily, high‐quality protein intake and intake of key micronutrients. More targeted nutrition approaches that promote muscle protein synthesis include amino acid supplementation with leucine and its metabolite β‐hydroxy β‐methylbutyrate. Another potential target for support is the gut microbiome, as its adequate function is increasingly seen as playing a role in human health and metabolism. Obesity is a heterogenous disease, and there is considerable interest in specific metabolic phenotypes that might be used to tailor nutrition strategies. As research advances on these and other fronts, there is the potential to identify precision nutrition strategies for individualized, more effective approaches to lifelong obesity management.

**Study Importance**

**What is already known?**

- To our knowledge, this is the first review to address strategies for multidisciplinary care and precision nutrition for patients with obesity in an era of increasingly effective treatments.

**What does this review add?**

- This article emphasizes the importance of nutrition adequacy during obesity treatment to preserve skeletal muscle and bone while achieving reductions in body fat, while discussing promising avenues to precision nutrition with greater attention to supplements that support muscle and gut microbiome function.

**How might these results change the direction of research or the focus of clinical practice?**

- We highlight a range of promising therapeutic strategies to minimize the loss of vital body components, including skeletal muscle and bone, during the reduction in body fat that comes with effective obesity management.
- We address specific physiologic, psychosocial, and behavioral factors that impact treatment success and should be addressed by a multidisciplinary care approach for patients with obesity.

## INTRODUCTION

The understanding of obesity as a complex and chronic disease has been well established, most recently in a 2023 consensus statement from six leading organizations dedicated to the prevention and treatment of the disease. This statement acknowledged not only the multisystem impact of obesity but also the need for an individualized approach to diagnosis and treatment that considers an individual's social determinants of health, age, race, and ethnicity, as well as the impact of potential societal bias on treatment access and health outcomes [1].

With this understanding of obesity as a chronic disease comes an evolution in treatment strategies that include effective obesity medications (OMs) in addition to lifestyle modifications and surgical options, as well as greater research attention to the role that nutrition plays in supporting health during weight reduction and maintenance. For instance, the weight reduction achieved with OMs and other interventions can impact not only excess adiposity but also lean tissues such as skeletal muscle, and strategies are needed to prevent excessive losses of these vital body components during weight reduction [2, 3]. The goal of establishing and maintaining nutrition adequacy during obesity treatment that prevents this lean mass loss is challenging and requires individualized approaches to optimize nutritional intake during weight reduction. Obesity treatment also carries the risk of weight cycling (WC) [4], a phenomenon of repeated episodes of weight reduction followed by regain, which can impact lean tissues such as skeletal muscle [5, 6, 7] and is often driven by physiologic, psychosocial, or behavioral factors, which clinicians need to understand and address through a multidisciplinary approach. Finally, innovative avenues in obesity research are now considering the influence of the gut microbiome [8] and precision nutrition strategies [9, 10, 11] to better address the chronic impact of obesity and its treatment through weight reduction.

Recognizing the global chronic health threat that obesity poses, the 122nd Abbott Nutrition Research Conference invited leading clinicians and scientists in October 2024 to discuss the evolving understanding of the multifactorial interplay of nutrition and body composition in obesity management. This article is a result of their insight and discussions, the content of which met continuing education criteria of being evidence based, fair and balanced, and nonpromotional. Of note, this article provides summaries of important topics in this area, but the chosen topics, conclusions, and recommendations presented here are the result of extensive collaborative review by the authors and not based on a more systematic consensus process.

## LEARNINGS FROM PAST TO PRESENT: MULTIDISCIPLINARY OBESITY CARE

Despite a wealth of modern clinical evidence linking obesity to a multitude of biochemical and environmental factors responsible for weight control, there is a long history of bias regarding the psychosocial impact of obesity risk and the contribution of individual factors, such as willpower, in lifelong obesity management. For instance, as recently as 2016, a survey of 1509 US adults found that three‐quarters of respondents believed that obesity stemmed from a lack of willpower [12]. Historically, researchers proposed links across obesity, social class, and contemporary markers of mental health [13]. Obesity was also seen as a problem remedied solely through the ability to control overeating or adhere to behavioral interventions [14]. Weight bias continues to be pervasive among medical professionals as well, but obesity diagnosis and referral for interventions may improve with targeted education interventions [15].

### Neuroendocrine network signaling, nutrient cues, and obesity

More recently, interactions between metabolism and endogenous hormones produced by the gut and/or adipose tissue, such as leptin [16, 17], have been recognized as important physiologic factors in the development of obesity. Caloric restriction has also been found to promote hormonal counter‐regulatory mechanisms involving the gastrointestinal and central nervous system [17]. Present understanding of this specialized endocrine network recognizes the influence of gastrointestinal hormones on nutrient cues and metabolic homeostasis [18].

### The need for a comprehensive approach to obesity care

**FIGURE 1:**
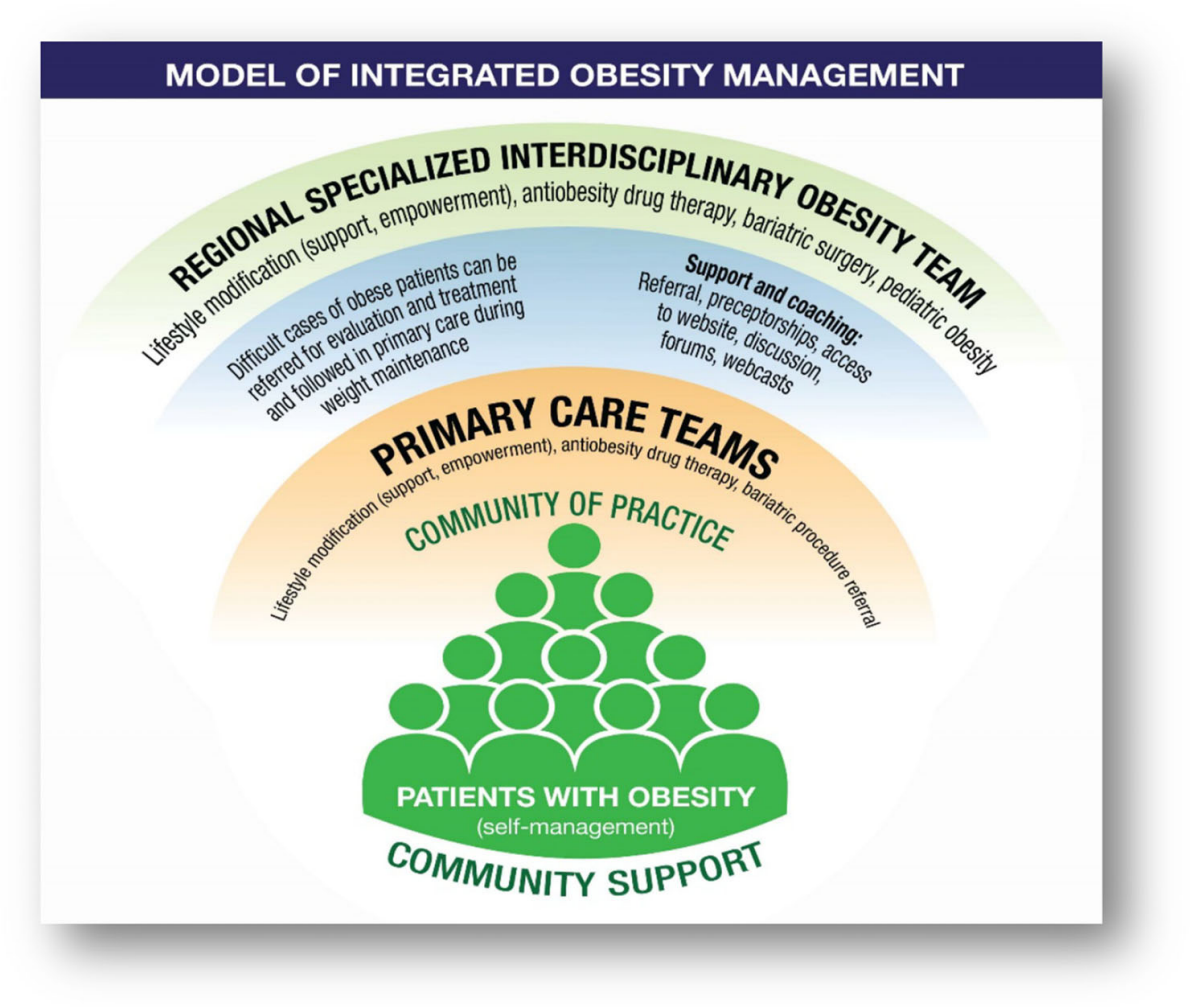
Model of integrated obesity management. Adapted from Baillargeon et al. [19].

Although the science of neuroendocrine biology continues to advance, overcoming the underlying pathophysiology in obesity is complex and requires a dedicated multidisciplinary approach to care. Contemporary treatment strategies continue to be founded on lifestyle modifications but are now complemented by pharmacotherapy and/or surgery as warranted. These interventions may be effectively delivered by one provider or a dedicated team of providers that deliver different aspects of evidence‐based, comprehensive treatment. This includes not only primary care providers but also registered dietitians, nutritionists, exercise physiologists, behavioral health specialists, pharmacists, and, for medically complex obesity cases, an obesity medicine specialist (Figure 1) [19]. Most patients with obesity are initially managed by primary care providers, and one program has aimed to improve these providers' efficacy in treating these patients through a targeted, multidisciplinary approach based on a chronic care model that better integrates care. This required an obesity education intervention for primary care providers [19] that may apply to other systems‐level interventions to improve obesity care. Knowledgeable primary care providers, including physicians, nurses, advanced practice nurses, pharmacists, and other clinicians responsible for primary care delivery, can effectively deliver support for lifestyle modifications, OM prescriptions, and referrals for more advanced care. This can include interventions from more specialized members of a multidisciplinary obesity care team, including bariatric surgeons and pediatric obesity professionals. The following topics will address specific aspects of comprehensive care and the continuing research that will better determine the individualized approaches to address the needs of the growing population of patients affected by obesity.

## PRESCRIBING OMs: BEYOND WEIGHT REDUCTION

Most pharmacotherapy strategies currently approved by the US Food and Drug Administration (FDA) for obesity management reduce appetite [20], acting either through central or neurometabolic pathways involving enteroendocrine and endo‐pancreatic hormones [21, 22, 23, 24, 25, 26, 27, 28, 29]. Recently introduced incretin mimetic‐based OMs have been highly effective in achieving weight reduction and will be the focus of this discussion.

### OMs and weight reduction

Semaglutide, a glucagon‐like peptide‐1 (GLP‐1) agonist, and tirzepatide, which acts as both a GLP‐1 and glucose‐dependent insulinotropic polypeptide agonist, are both FDA‐approved OMs that have demonstrated substantial weight reduction in placebo‐controlled trials. In the randomized, placebo‐controlled Semaglutide Treatment Effect in People with Obesity (STEP) 3 trial, subcutaneous semaglutide 2.4 mg once weekly in addition to intensive behavioral therapy in patients with overweight or obesity was associated with 16.0% weight reduction versus 5.7% with placebo (*p* < 0.001). Of note, the addition of intensive behavioral therapy in STEP 3 only achieved an additional 1% weight loss compared with weight loss achieved with semaglutide in addition to a less‐intensive lifestyle intervention in the STEP 1 trial [30]. The Study of Tirzepatide (LY3298176) in Participants After a Lifestyle Weight Loss Program (SURMOUNT)‐3 randomized, placebo‐controlled trial reported that in patients with overweight or obesity and at least one obesity‐related complication (excluding diabetes) who had initially achieved a ≥5.0% weight reduction with an intensive lifestyle intervention, tirzepatide resulted in an additional 18.4% weight reduction versus 2.5% weight gain with placebo (*p* < 0.001) [31].

Further analyses from the STEP clinical trial program have reported positive effects on dietary behavior. In the STEP trials, when compared with placebo, semaglutide resulted in significant improvements in all domains of the Control of Eating Questionnaire, including craving control, craving for savory, craving for sweet, and positive mood [32]. Tirzepatide has also been associated with improved eating behaviors in patients in Japan with type 2 diabetes [33].

As the field of OMs expands, researchers continue to explore the effects of incretin mimetic‐based OMs acting on GLP‐1, glucose‐dependent insulinotropic polypeptide, and other hormone receptors, as well as novel agents that act on multiple receptors, in the hope of better targeting the complex endocrine‐based pathophysiology of obesity disease [34].

### Cardiometabolic impact of OMs

The body weight reduction achieved with second‐generation OMs, namely semaglutide and tirzepatide, is considerable and has been associated with positive impacts beyond weight reduction, especially on important biomarkers of cardiovascular health and tissue inflammation [35]. The potential for these OMs to exert beneficial anti‐inflammatory tissue effects, in addition to beneficial impacts on blood pressure, platelet aggregation, and insulin resistance, is supported by evidence of reduced cardiometabolic risk. For instance, tirzepatide has been shown in a meta‐analysis to result in clinically meaningful reductions in blood pressure, low‐density lipoprotein cholesterol, and triglycerides, as well as an increase in high‐density lipoprotein cholesterol [36]. In the same analysis, tirzepatide was likewise shown to improve hemoglobin A1c and reduce overall type 2 diabetes risk [36]. A phase 3, randomized, placebo‐controlled trial in patients with type 2 diabetes found that semaglutide, when compared with placebo, was associated with significantly lower rates of cardiovascular mortality, nonfatal myocardial infarction, or nonfatal stroke [37]. More recently, in a larger multicenter, double‐blind, placebo‐controlled trial in patients with preexisting cardiovascular disease and overweight or obesity, but no history of diabetes, semaglutide 2.4 mg once weekly was associated with a significant reduction in a composite cardiovascular endpoint of death from cardiovascular causes, nonfatal myocardial infarction, or nonfatal stroke (*p* < 0.001) at a mean follow‐up of 39.8 months [38].

### OMs and other health outcomes

Tirzepatide and semaglutide have also demonstrated benefits beyond cardiovascular risk reduction and improvements in metabolic health that have resulted in an expanded FDA‐approved indication for semaglutide 2.4 mg for cardiovascular risk reduction in at‐risk patients with overweight or obesity. In December 2024, tirzepatide became the first FDA‐approved medication for obstructive sleep apnea based on phase 3 results from the SURMOUNT clinical trial program [39]. The STEP research program continues to evaluate the wider benefits of the weight reduction seen with semaglutide, including sustained weight reduction and maintenance over 2 years in the STEP 5 trial [40]. Other areas of research focus in the STEP program include the potential of semaglutide 2.4 mg once weekly to positively impact quality of life, obstructive sleep apnea, osteoarthritis, kidney dysfunction, and depression, all of which are substantially impacted in patients with obesity [32].

### The importance of lean mass preservation during obesity management

The term lean body mass is sometimes used to describe the mass of skeletal muscles and bones, whereas fat‐free mass (FFM) is a more inclusive term that also accounts for other nonfat components like organs and blood [41]. Obesity is associated with higher baseline FFM and skeletal muscle mass (SMM), and intentional weight reduction results in greater reductions in body fat over FFM or SMM, as well as improvement in overall muscle quality [3]. Although reductions in overall lean mass with intentional weight loss have been observed, there is some evidence that this loss is primarily low‐density or low‐quality muscle mass, with a preservation of normal density muscle [3]. The weight reduction achieved with OMs is not thought to cause physical frailty or progressive muscle mass loss (sarcopenia) [2].

As obesity management continues to be impacted by the emergence of OMs for weight loss through restricted energy intake, it is important to consider the relative ability to preserve lean mass solely through intake restriction versus restriction that includes physical activity. Despite the importance of this topic, there is a lack of evidence describing changes in physical activity or cardiovascular fitness in response to weight loss due to reduced dietary intake. A review on the role that lifestyle modification plays in conjunction with newer, second‐generation OMs acknowledges the importance of physical activity, including activities focused on muscle strengthening, especially in older adults. Indeed, lean mass and SMM loss with rapid weight reduction through OMs across the age spectrum is an ongoing concern that requires further research [3].

Some lean mass can be lost during weight reduction, which can in turn result in decreased bone mineral density, insulin sensitivity, and aerobic capacity [3]. A review of current evidence suggests that to protect against SMM loss during intentional weight reduction with dietary energy restriction through treatment with newer OMs, individuals should aim to consume 0.8 g/day of protein per kilogram of body weight or at least 60 to 80 g/day of high‐quality protein, with some individuals requiring higher amounts than this (e.g., those undergoing bariatric surgery) [3]. Older adults also have higher protein requirements and should aim for a dietary intake of 1.0 to 1.2 g/kg protein per day and patients with chronic disease require 1.2 to 1.5 g/kg of protein daily [42]. However, these are broad recommendations and guidance on protein intake should be prescribed on an individual basis. Based on public health guidelines, the review further recommends that individuals treated with these OMs should engage in regular physical activity that includes aerobic activity, muscle strengthening, and balance training [3]. One randomized, placebo‐controlled study found that liraglutide in combination with a moderate‐to‐vigorous exercise program was more effective in maintaining healthy weight loss compared with either intervention alone [43]. In addition, a 2015 guideline from the Endocrine Society stresses the importance of diet, exercise, and behavioral intervention in addition to pharmacologic therapies used to achieve weight reduction [44]. With the important caveat that evidence is currently limited, these lifestyle recommendations may help reduce the risk of lean mass loss during obesity treatment. Scant evidence is likewise available regarding nutritional adequacy in patients taking OMs as these medications impact hunger and satiety and could likewise make recommended dietary intake difficult to achieve consistently [45, 46]. Thus, long‐term supervised care with a health care professional is critical to monitor nutrition intake and quality. As additional evidence emerges, precision nutrition approaches that consider supplementation or targeted dietary interventions may also play a role in this setting to ensure long‐term nutritional adequacy. It is generally established that energy restriction (voluntary or medication driven) also induces moderate bone loss [47]. GLP‐1 agonists are likely to mimic this effect [48]. The impact of exercise and dietary protein may facilitate mitigation of bone loss [49].

Finally, it is important to recognize the risk of sarcopenia in patients with obesity, which in this context is referred to as sarcopenic obesity. A joint guideline from the European Society for Clinical Nutrition and Metabolism and the European Association for the Study of Obesity suggests that patients who present with markers of low SMM based on clinical symptoms or validated questionnaires, in addition to elevated body mass index (BMI) or waist circumference, should be considered at risk for sarcopenic obesity and undergo diagnostic assessments of SMM and skeletal function. Recommended assessments include markers of skeletal muscle function, including handgrip strength, knee‐extensor strength, and chair stand test, as well as measures of body composition with dual energy x‐ray absorptiometry (DXA) or bioelectrical impedance analysis when DXA is not feasible [41]. The Sarcopenia Definition and Outcomes Consortium has further clarified that sarcopenia should also include measures of low grip strength and low usual gait speed [50].

As nutrition is known to exert a powerful influence on obesity management and the preservation of critically essential muscle mass during weight reduction [3], the following sections will focus on maximizing nutrition in patients with obesity and innovative strategies to support muscle health, including supplementation, gut microbiome support, and precision nutrition.

## MAXIMIZING NUTRITION IN OBESITY TREATMENT: LESSONS FROM BARIATRIC SURGERY

**FIGURE 2:**
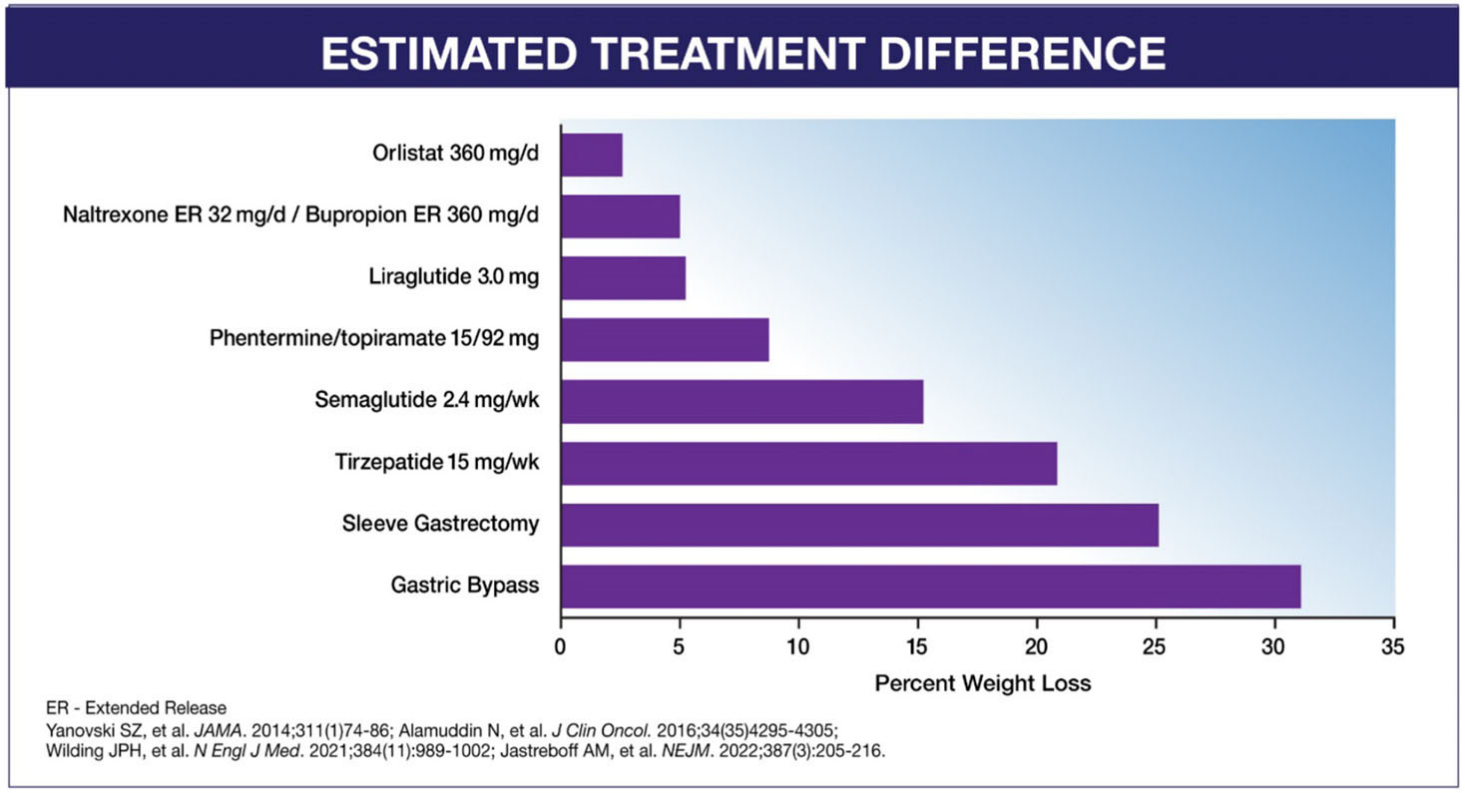
Estimated percentage of excess weight loss compared with placebo achieved with available medication and surgical treatment options for obesity. Excess weight loss compared with placebo is the weighted mean difference for the drug‐to‐placebo comparison for the respective drug. Observed excess weight loss was observed at 52–72 weeks of treatment. [51, 52, 53, 54].

When comparing the relative impact of available interventions on weight reduction in patients with obesity, surgical procedures such as gastric bypass and sleeve gastrectomy achieve the greatest degree of weight reduction, followed by newer OMs, including semaglutide and tirzepatide as previously discussed, as shown in Figure 2 [51, 52, 53, 54].

The Obesity and Weight Management for the Prevention and Treatment of Type 2 Diabetes: Standards of Care in Diabetes–2025 guidelines from the American Diabetes Association recommend that initial treatment for overweight and obesity with lifestyle and nutritional therapy, pharmacologic agents, or metabolic surgery should be individualized to reflect “the person's medical history, life circumstances, preferences, and motivation” and that a combination of these approaches could be considered appropriate in higher‐risk individuals [55]. Tirzepatide and semaglutide have both demonstrated sustained weight reduction when added to behavioral interventions in placebo‐controlled trials [30, 31]. The GLP‐1 agonist liraglutide has also been associated with effective weight reduction when used in conjunction with behavioral interventions in placebo‐controlled trials [56, 57].

### Comparing nutrition intake after bariatric surgery or treatment with OMs

Although findings are not consistently conclusive, initial research suggests that early dietary intervention through registered dietitian support may improve the likelihood of effective weight loss after bariatric surgery [58, 59, 60]. However, long‐term contact with a registered dietitian has not been associated with lasting behavioral change, and initial follow‐up visits are more likely to focus on symptom management and malnutrition prevention [58]. Overall, calorie intake is significantly reduced in the first 6 months after surgery (45%–75%) but tends to increase, with a reduction of 19% to 50% at 1 year. Although no standardized nutritional recommendations have been issued that are specific to bariatric procedures, the reported initial caloric intake after surgery of less than 800 calories per day in most patients is insufficient to adequately meet an individual's daily nutritional needs [58].

Patients undergoing obesity treatment with OMs with the goal of weight reduction have reported reductions in caloric intake of 16% to 39% [45]. Although scant evidence is available on intake after initiating OMs, patients should be assessed for any existing nutritional risk factors and receive counseling on how to maintain an adequate intake of protein, dietary fiber, micronutrients, and fluids prior to the initiation of treatment. Once treatment is initiated, patients should be monitored for gastrointestinal symptoms or inadequate nutritional intake [46].

### Macronutrient recommendations

As noted earlier, patients undergoing bariatric surgery are advised to aim for a daily protein intake of at least 60 g/day and up to 80 g/day. A review of available nutritional data from patients who underwent bariatric surgery found no relationship between macronutrient intake distribution and weight reduction, with reported intakes of 35% to 50% from carbohydrates, 15% to 23% from protein, and 35% to 42% from fat [59]. For patients receiving OMs, clinicians should recommend daily intakes of 45% to 65% from carbohydrates (130 g/day) and 20% to 35% from fats, with the potential for daily protein intake of at least 60 g/day [46].

### Recommended micronutrient management

Patients who undergo bariatric surgery should be supported with long‐term follow‐up care that includes the routine monitoring of micronutrient status. Up to 10% of micronutrient intake may be poorly absorbed after bariatric procedures, but deficiencies often arise due to reduced intake and rapid weight reduction. To account for potential micronutrient deficiencies, patients are commonly prescribed supplements including iron, calcium, vitamin D, and B vitamins [59]. Attention to the potential for deficiencies through provision of nutrient‐dense foods, fortified foods, and/or dietary supplements is supported by evidence that, regardless of treatment strategy, caloric reduction may exacerbate nutritional inadequacy, as evidenced in one analysis based on data from the National Health and Nutrition Examination Survey 2015–2018 [61]. Although limited evidence is available on micronutrient intake or potential deficits after initiation of OMs, clinicians may consider supplementation with a multivitamin, calcium, or vitamin D, with ongoing monitoring of micronutrient status as warranted [46].

### Symptom‐related nutrition issues

Gastrointestinal symptoms that could affect nutritional intake are common with bariatric surgery and OMs. Up to 80% of patients undergoing bariatric surgery experience nausea or vomiting, and postoperative changes in bowel habits, including both diarrhea and constipation, are also common [62]. Nausea and vomiting have both been commonly reported with OMs, including semaglutide and tirzepatide, with reported nausea rates in phase 3 studies considerably lower than rates seen with bariatric surgery (44.2% and 31.0% with semaglutide and tirzepatide, respectively) [53, 54].

### Other nutritional considerations from bariatric surgery experience

As OMs are increasingly used to manage obesity, there are important lessons to be learned from bariatric surgery, including the importance of patient engagement of education, careful attention to potential of weight bias, direct plain‐language communication, and recognition of accessibility challenges that patients may face. Future research should better characterize the role of diet quality in recovery and weight maintenance, micronutrient monitoring, and identification of patients who need a higher level of care.

## THE PSYCHOLOGICAL, PHYSICAL, AND METABOLIC CONSEQUENCES OF WC

WC, or intentional weight reduction followed by weight regain, which is sometimes referred to as the yo‐yo effect in lay terms, is a consequence of repeated episodes of striving for reduced body weight and can affect individuals in all BMI categories. The causes of WC can include hypocaloric eating that results in hunger and lack of satiety, withdrawal of OMs, or a history of disordered eating behaviors [4]. This phenomenon can be particularly impactful in patients with obesity, especially as WC has been associated with a negative impact on metabolic health, especially in female individuals [4, 6]. WC rates are not only higher in female than in male individuals [4], but female individuals with a history of WC are more likely to exhibit adverse markers of cardiometabolic health, including higher lipids and Homeostasis Model Assessment of Insulin Resistance (HOMA‐IR), than female individuals without a history of WC [6].

WC can result in important physiologic changes, including increased visceral and subcutaneous fat accumulation, decreased lean mass (especially SMM), frequent fluctuations in cardiovascular risk markers, and systemic inflammation [5, 6, 7]. The impact of WC on body composition is more prominent in women, resulting in the loss of muscle mass and function as well as increased body fat accumulation. These changes in body composition promote inflammation, which adversely impacts the ability to reduce body fat and build muscle mass. WC also increases the risk of multiple comorbid diseases, including obesity, type 2 diabetes, hypertension, cancer, bone fractures, sarcopenic obesity, eating disorders, and psychological conditions [63].

### Psychological impact of WC

The long‐term psychological impact of WC can be considerable, especially in patients with obesity, and eating behavior has been found to be a potential driver of psychological distress in those who experience WC. In a study of a 3‐month outpatient intervention of diet and exercise for 153 patients with a history of WC, psychological distress was found to have a direct effect on eating behavior (3 months, β = 0.181, 95% CI: 0.055–0.310; 6 months, β = 0.182, 95% CI: 0.039–0.332). Psychological distress was found to have a bidirectional impact on weight control and was found to impact eating behavior as well [64]. Psychological distress in patients with obesity and a history of WC may be linked to anxiety, depression, lack of body confidence, or body image anxiety related to societal stigma [64, 65, 66].

### Strategies for WC prevention

Strategies for WC prevention are founded on the recognition of obesity as a chronic, progressive, relapsing disease and the need to manage those affected accordingly with patient‐centered interventions. This includes a focus on adiposity reduction rather than weight reduction, a prioritization of retaining muscle mass and function during treatment, sustainable eating and exercise patterns that promote body fat reduction with muscle and skeletal mass retention, and the use of OMs and other pharmacotherapy strategies as appropriate.

### Nutritional strategies to maintain muscle mass

Eating patterns that promote fat reduction while maintaining muscle mass include ensuring adequate protein intake; prioritizing vegetables, fruit, and legumes; limiting processed carbohydrates; and eating frequent small meals to avoid excessive hunger [67, 68]. Intake of dietary amino acids, especially essential amino acids, has a positive effect on muscle protein synthesis (MPS). The efficiency of protein synthesis declines in older adults, resulting in higher protein needs for older adults than what is recommended for healthy adults. Although guidelines recommend a daily protein intake of 0.8 g/kg body weight in healthy young adults, higher daily protein intakes are recommended to maintain muscle mass and function in individuals 65 years of age and older (1.0 to 1.2 g/kg) and 1.2 to 1.5 g/kg in patients with chronic diseases [42]. These higher protein recommendations may be especially important for older adults who are affected by obesity.

Of note, the current literature on protein intake and its impact on muscle preservation and synthesis has focused on older healthy adults [42, 67, 69, 70, 71]. It is still unclear whether these findings can be consistently applied to patients with obesity across the age spectrum, but there are important aspects of protein intake that may be considered as clinicians help patients with obesity identify tailored nutritional strategies that support muscle mass preservation. For instance, a narrative review of exercise and nutritional strategies to combat sarcopenia stresses that the type and timing of daily protein intake is important. Protein derived from animal sources, including whey protein, may be more effective at promoting muscle preservation and synthesis [72]. Whey protein enriched with the amino acid leucine and vitamin D has been shown to enhance postprandial MPS in healthy older men [69]. In terms of protein intake timing, an even protein intake of every 3 to 4 h may more effectively promote muscle strength, physical performance, and SMM in older adults [70]. One review showed that a total protein intake of 1.2 to 1.6 g/kg per day, which may potentially include meal‐specific protein quantities of 25 to 30 g, provides improvements in appetite, body weight, and cardiometabolic risk factors. However, the authors concluded that research is needed to determine meal‐specific protein quantity and timing [73]. Finally, the nutritional impact of protein intake is also affected by physical activity, which includes muscle strengthening and aerobic activity [71]. These findings are promising and may be considered as clinicians and patients develop nutritional strategies during intentional weight loss, but further research on protein intake and lean mass preservation in patients with obesity is critical. Any discussion of nutrition must also consider numerous geographic and socioeconomic factors that impact individuals' ability to achieve adequate, high‐quality nutrition in the United States. According to the US Department of Agriculture Food Access Research Atlas, 17.4% of the US population lives in areas that are low income and vulnerable to low‐food access, with travel of up to 10 miles needed to reach the nearest supermarket [74].

Targeted nutrition is important for better outcomes in patients with obesity, and the remaining topics will highlight important aspects of this approach, including amino acid supplementation, the role of the gut microbiome in obesity, and modern precision nutrition strategies.

## COULD β‐HYDROXY β‐METHYLBUTYRATE BE A KEY PLAYER TO SUPPORT MUSCLE HEALTH DURING WEIGHT REDUCTION?

Skeletal muscle plays an important role in aging and disease, and the preservation of SMM is an important treatment goal for patients with obesity. SMM accounts for approximately 50% of body mass and is responsible for physical movement, physical strength, posture, and balance [75]. Decreased SMM or function due to aging, orthopedic injuries, or disease contributes to the risk of certain cancers, chronic obstructive pulmonary disease (COPD), diabetes, obesity, arthritis, and hospitalizations [75, 76].

As previously discussed, the timing of protein intake has the potential to impact the efficiency of muscle support through MPS. In one study in healthy men, a protein meal had a transient effect on MPS [77]. Another study found that muscle disuse due to inactivity results in suppressed MPS in response to nutritional protein intake [78].

To study the impact of MPS on overall muscle preservation, amino acid supplementation that promotes MPS has been investigated. Specifically, the amino acid leucine has demonstrated a potent ability to promote MPS [79] and therefore has been researched, in addition to its metabolite β‐hydroxy β‐methylbutyrate (HMB), as a potential nutritional supplement to support MPS. Although the effects of HMB on acute human muscle protein turnover are still unclear, HMB has been shown to stabilize muscle cell membranes, reduce protein catabolism, and increase MPS. A 3‐week placebo‐controlled study in healthy men also reported that HMB demonstrated evidence of increases in both lean mass and muscle strength compared with placebo [79, 80].

### Research evidence supporting HMB supplementation for muscle mass and strength

A systematic review and meta‐analysis confirmed that nutritional supplementation with HMB may improve SMM (measured through DXA or bioelectrical impedance analysis) and physical function in patients with a variety of clinical conditions [81], and multiple randomized trials have explored potential benefits in hospitalized patients and older adults. In a randomized, controlled, double‐blind, parallel‐group study of otherwise healthy adult hospitalized patients, nutritional supplementation that contained HMB improved lean mass preservation during bed rest, as measured by DXA [82]. Another randomized study reported that supplementation that contained HMB prevented muscle catabolism, as determined by reductions in blood urea nitrogen, in elderly bedridden patients receiving tube feeding [83]. Additional randomized trials reported improved SMM retention, as determined by DXA, and handgrip strength in patients who received HMB supplementation after liver transplantation [84]. HMB‐supplemented oral nutritional supplements also reduced mortality risk and improved handgrip strength in a study of hospitalized patients with COPD [85]. This randomized study of 7 days of HMB supplementation in patients with COPD in the intensive care setting reported improved pulmonary function, reduced inflammation, and evidence of reduced protein catabolism as measured by creatinine and total protein values [86]. In healthy older adults, HMB supplementation has been shown to improve physical strength, FFM, and lean mass, measured by DXA in multiple studies [87, 88, 89, 90].

### The role of HMB in patients with obesity

As previously discussed, clinicians treating patients for obesity are challenged with the task of maintaining muscle mass and function while achieving adiposity reduction. The availability of newer OMs has reinforced the need for strategies that maintain lean mass during weight reduction. For instance, in one randomized study, weight reduction achieved with a very low‐calorie diet alone or in conjunction with the OM semaglutide resulted in reduced lean mass in addition to weight reduction [91].

Research to date has not investigated HMB supplementation to address lean mass preservation during weight reduction in patients with obesity through robust, placebo‐controlled studies. Nevertheless, HMB supplementation has received interest in other settings in which individuals experience weight reduction. For instance, a placebo‐controlled, randomized study in college‐aged male boxers experiencing acute weight reduction found that HMB supplementation showed evidence of preserving FFM [92]. Another randomized, placebo‐controlled study found that 6 weeks of HMB supplementation increased muscle strength in sedentary older women who had overweight [93]. Ultimately, the effects of HMB on muscle protein turnover, lean mass, and strength preservation in humans undergoing weight reduction are still unclear, and additional research is needed to characterize the extent and consistency of effects seen in initial studies.

Muscle plays a central role in strength, mobility, and metabolism, and the loss of muscle mass leads to significant negative health outcomes across the continuum of care. Clinicians should recognize the risk of muscle mass loss during weight reduction in patients with obesity and consider supplementation when warranted, in addition to nutrition and physical activity, to retain muscle mass and function. Supplements that include HMB and other targeted nutrition strategies may further support this goal.

## THE INTERPLAY OF NUTRITION, OBESITY, AND THE GUT MICROBIOME

The gut microbiota or microbiome represents a cell‐rich and complex community of hundreds of microbial species, including bacteria, fungi, archaebacteria, viruses, and protozoans [94]. This community is metabolically active, influenced by diet, and responsible for critical physiologic processes in the human body, including immune function, nutrient acquisition and absorption, and resistance to infections [95]. Directly relevant to obesity are findings that anaerobic carbohydrate fermentation producing short‐chain fatty acids (SCFAs) provides an estimated 5% to 10% of human energy requirements [96]. In addition, the gut microbiota produces metabolites (e.g., SCFAs, bile acid derivatives, tryptophan‐derived aryl hydrocarbon receptor agonists [e.g., indole‐3‐propionic acid], which have metabolic, immunomodulatory, and anti‐inflammatory effects and stimulate hormones [e.g., GLP‐1, PYY]) [97]. SCFAs do also induce mucus production on the intestinal epithelium, while degradation of the mucus layer by gut microbes has been shown to lead to a reduction of intestinal epithelial integrity and inflammation and ultimately pathology in animal models of obesity, type 2 diabetes, and metabolic syndrome [98]. Research that compared colonized with axenic (germ‐free) animals has demonstrated a causal contribution of the gut microbiome to pathologies in animal models of obesity, type 2 diabetes, and metabolic syndrome [99, 100, 101]. It is difficult to make such causal inferences in humans [102], but microbiomes are altered in individuals with obesity [103, 104], and proof‐of‐concept studies have shown that insulin sensitivity can be improved in individuals with metabolic syndrome using fecal microbial transplantation (FMT) from healthy lean donors [105, 106, 107]. Although the mechanisms of gut microbiome effects are complex and not yet fully understood, gut microbiome composition and dysfunctional metabolic activity are likely to contribute to obesity and its comorbidities [95].

### The microbiome as a target for therapeutic strategies for obesity management

A variety of strategies are now under consideration to improve gut microbiome composition and metabolic function to support health and manage obesity and its comorbidities. As already discussed, FMT has been explored in individuals with obesity and metabolic syndrome. One proof‐of‐concept study demonstrated that a single‐dose oral FMT from healthy lean donors combined with a daily low‐fermentable fiber supplementation produced small but statistically significant improvements in insulin sensitivity in patients with severe obesity and metabolic syndrome [105]. Live biotherapeutics are under investigation, as are probiotic, prebiotic (fiber), and synbiotic supplements. Such strategies can be devised to enhance SCFA production, enhance microbiome metabolism, induce host hormones and mucus production, and reduce mucus degradation, and in animal models, they have been shown to improve pathologies linked to obesity [108, 109]. Some of these approaches have also shown promising results in humans [110, 111], but effect sizes in clinical trials have been small, and no gut microbiome targeted approach has obtained regulatory approval for the treatment of obesity. Despite promising research avenues, the mechanisms of gut microbiome effects on human health and disease have yet to be fully elucidated, and current evidence is variable and limited.

### The microbiome as a target for nutritional strategies for obesity management

Dietary approaches, especially whole diets rich in plant‐based foods such as vegetables, fruits, legumes, and nuts, such as the Mediterranean diet, are known to improve overall health and play an important role in obesity prevention and management. There is increasing evidence that at least some of these effects are mediated through the microbiome [112]. Whole food diets with adequate fiber intake improve gut microbiome functions (e.g. enhanced fermentation, lower luminal pH, promotion of SCFAs, inhibition of proteolytic fermentation) and host‐microbiome interactions (e.g. at the epithelial interface) that are relevant for obesity, for example by reducing mucus degradation [112, 113, 114]. In contrast, food components of highly processed foods, such as emulsifiers, disrupt the mucus layer and are thought to contribute to inflammation [115]. A recent study evaluated a diet that recapitulated key characteristics of nonindustrialized dietary patterns (primarily plant‐based, limited in highly processed foods, and with 22 g of dietary fiber per 1000 kcal) in a randomized controlled feeding trial in healthy adults. Three weeks on this diet increased microbiome fermentation, reduced genes in the fecal microbiome that encode for enzymes that degrade mucus, and beneficially altered microbiota‐derived plasma metabolites implicated in the etiology of obesity (e.g. an enrichment of indole 3‐propionic acid, a metabolite linked to lower risk in type 2 diabetes). Although adjusted to maintain individual caloric requirements, the diet caused a significant weight reduction and considerable cardiometabolic benefits (17% reduction in plasma fasting low‐density lipoprotein, 6% reduction in fasting glucose, and 14% reduction in C‐reactive protein). Several of these effects were linked to baseline and diet‐responsive microbiome features with established roles in obesity pathology (e.g., fermentation capacity, SCFAs, *Bilophila*, mucus‐degrading genes) [116].

Overall, these findings echo similar convincing findings with other plant‐rich diets (e.g. the Mediterranean diet), support the substantial value of improving dietary habits in obesity prevention and management, and consider the gut microbiome's role in the effects of diet [112]. The effect sizes of the cardiometabolic benefits of such diets in published trials are often quite large and rather consistent among patients who are not yet prediabetic, supporting the value of population‐based dietary guidance. However, the gut microbiome is highly individualized, and individuals present with different clinical challenges and complications, including distinct metabolic phenotypes. Thus, if the response to a microbiome targeted approach is variable, machine learning and artificial intelligence approaches using microbiome, metabolic phenotypes, and clinical evaluations may play a role in a personalization of dietary and therapeutic strategies for obesity management. If such personalized approaches also include the dietary preferences of individuals, they may further improve adherence to diets and might thus result in better outcomes.

## PERSONALIZED AND PRECISION NUTRITION IN THE MANAGEMENT OF OBESITY

There is a growing recognition of the need for precision nutrition to address many conditions, including obesity and its associated comorbid metabolic diseases [10, 11]. Specifically, the term precision nutrition refers to tailored nutritional recommendations based on aspects of an individual's internal and external environments over the course of a lifetime, including genetic factors, dietary behaviors, food access, physical activity, and the microbiome [11]. As previously discussed, obesity is a complex disease and individual patients present with unique clinical challenges and complications, including distinct metabolic phenotypes, or metabotypes. One metabotype in patients with obesity exhibits tissue‐specific (i.e., liver and muscle) insulin resistance and has received research interest as a potential target for precision interventions in patients with obesity and metabolic disease [10].

**FIGURE 3:**
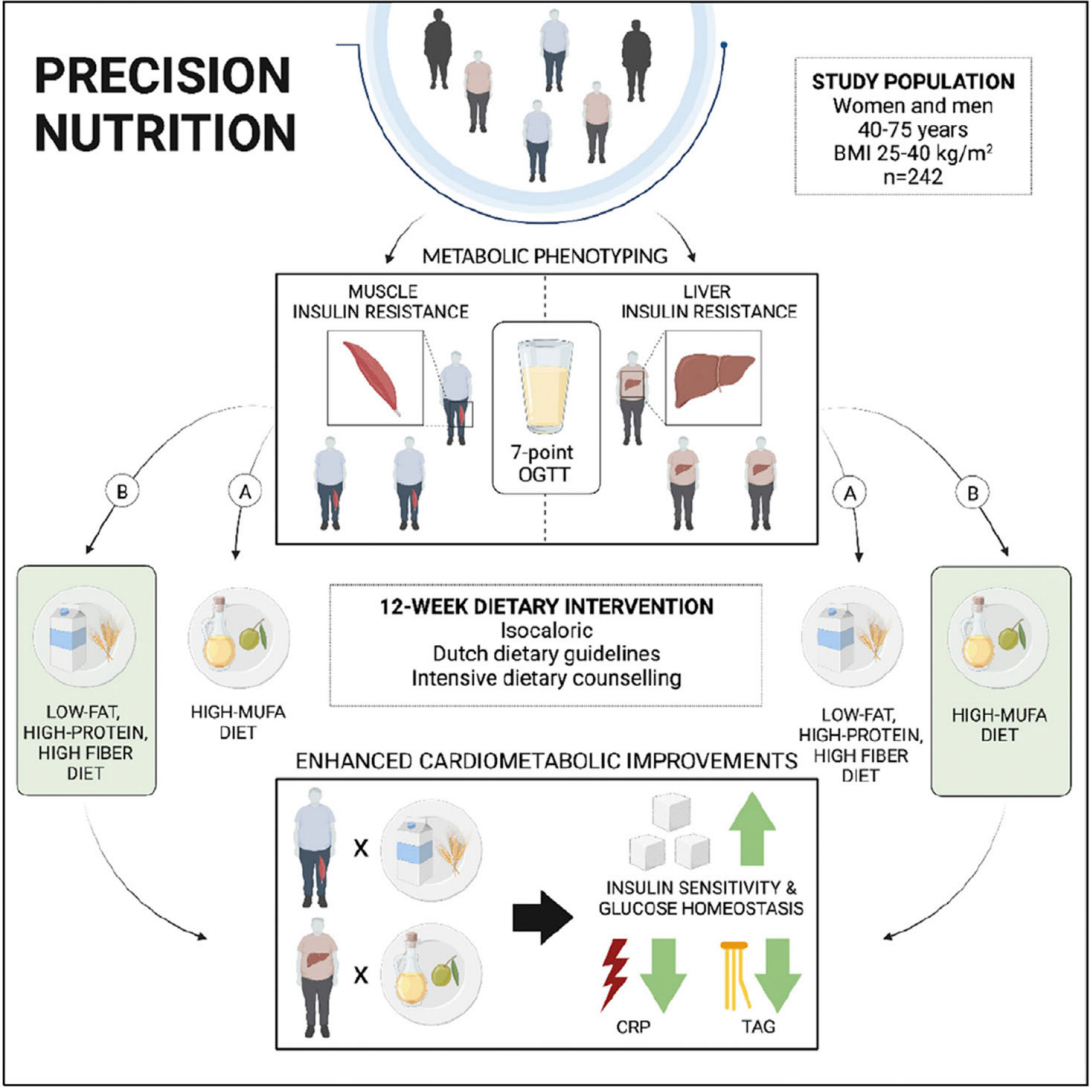
Investigated precision nutrition strategy based on insulin‐resistant metabotypes. Reprinted from Trouwborst et al. [9]. CRP, C‐reactive protein; MUFA, monounsaturated fatty acid; OGTT, oral glucose tolerance test; TAG, triacylglycerol.

Liver and muscle insulin resistance each displays distinct metabolic profiles and patterns of gene expression [117, 118, 119, 120], and both are under investigation as playing roles in innovative precision nutrition approaches in patients with obesity and other metabolic diseases. One study, summarized in Figure 3, evaluated precision nutrition centered on specific macronutrient provision based on the muscle insulin‐resistant or liver insulin‐resistant metabotype. Individuals were randomized to a low‐fat, high‐protein, high‐fiber diet or a diet high in monounsaturated fatty acids (MUFA). Researchers hypothesized that patients with the muscle insulin‐resistant metabotype would exhibit more metabolic benefit with a diet high in MUFA, whereas the liver insulin‐resistant metabotype would benefit from the low‐fat, high‐protein, high‐fiber diet. However, the study findings found the opposite trend, as patients with the muscle insulin‐resistant metabotype exhibited greater improvements in cardiometabolic health with a low‐fat, high‐protein, high‐fiber diet, whereas those with the liver insulin‐resistant metabotype exhibited greater cardiometabolic benefits with the high‐MUFA diet. Improvements in cardiometabolic health were measured by improvements in insulin sensitivity, glucose homeostasis, serum triacylglycerol, and C‐reactive protein. The authors noted that cardiometabolic benefits with the selected dietary macronutrient interventions were driven by insulin‐resistant phenotypes independent of weight loss. These findings suggest that a precision nutrition approach founded on metabolic phenotypes may be better than diets prescribed on general recommendations [9].

Important interactions have been found between gut microbiome function and human metabolism, including the expression of specific metabotypes in patients with obesity. For instance, as the success of specific dietary strategies may be influenced by individual metabotypes, the microbiome may be influenced through a relative balance between carbohydrate and protein fermentation [8]. Overall, baseline gut microbiota composition and functionality may be important determinants of outcomes with precision nutrition strategies to address cardiometabolic risk [106, 121, 122, 123, 124].

## CONCLUSION

Obesity is a complex disease and a global health concern that requires innovative strategies to achieve durable weight reduction and maintenance with a reduced risk of common obesity complications. The complexity and multifaceted nature of obesity, including the considerable psychological impact of the disease, suggest the need for a multidisciplinary approach that addresses individual patient lifestyle factors as well as prevents or minimizes recurring experiences of weight reduction and regain to prevent further cardiometabolic consequences. Although the treatment paradigm for obesity continues to be founded on lifestyle interventions that support nutrition and physical activity, durable weight reduction management is achieved with bariatric procedures and, more recently, a new generation of effective OMs. As patients experience weight reduction during treatment, nutrition plays an integral role in supporting health while reducing the risk of excessive muscle mass or function loss. Beyond the provision of adequate dietary protein and micronutrients based on patient needs and comorbidities, promising effects seen in initial studies suggest that supplementation with HMB may show potential for use in patients at risk for muscle loss, including those under treatment for obesity. The effects of HMB in patients with obesity, especially those receiving OMs that achieve rapid weight reduction, should be determined with additional research focus. Meanwhile, the gut microbiome and specific metabolic phenotypes are two areas requiring further research as the medical community continues its work to address obesity as a public health crisis. Current evidence suggests a future role for precision nutrition strategies that consider aspects of a patient's experience of obesity beyond a one‐size‐fits‐all approach to intervention.

## FUNDING INFORMATION

Funding for the virtual global conference was provided by Abbott Nutrition Medical Affairs and Research.

## CONFLICT OF INTEREST STATEMENT

Steven B. Heymsfield reports consulting fees and honoraria fees from Lilly, Tanita Inc., Regeneron, and Abbott. He has also received compensation for chairing an international meeting on nutrition and obesity management from Abbott Nutrition Medical Affairs and Research, serves on a safety data monitoring committee for Novo Nordisk, and is a board member for the Blackburn Foundation. Philip J. Atherton reports consultancy and honoraria fees from Abbott Nutrition. Sandra Christensen reports consultancy and honoraria fees from Abbott Nutrition, Novo Nordisk, Eli Lilly, the Obesity Medicine Association, the American Association of Nurse Practitioners, Advanced Registered Nurse Practitioners United of Washington State, Medical Learning Institute, Clinical Care Options/Practicing Clinicians Exchange, the University of Washington Cardiometabolic Extension for Community Healthcare Outcomes (ECHO) Program, and Forefront Collaborative. She has served on advisory boards for Abbott Nutrition, Novo Nordisk, and Eli Lilly. She is president and past trustee of the Washington Obesity Society and a trustee of the Obesity Medicine Association. Colleen Tewksbury reports consultancy and honoraria fees from Abbott Nutrition, Eli Lilly, TD Cowen, and the Commission on Dietetic Registration. She has received grants from the University Research Foundation and the Institute of Translational Medicine and Technology. She also serves as commissioner of the Commission on Dietetic Registration. Amanda Velazquez reports consultancy and honoraria fees from Abbott Nutrition Health Institute, the University of Chicago Academy for Continued Healthcare Learning, the Endocrine Society, and Elsevier. She has received support for meeting travel from The Obesity Society and has served on data safety monitoring committees or advisory boards for IntelliHealth, Eli Lilly, and WW International, Inc. (formerly Weight Watchers). She also serves on The Obesity Society Nominations Committee and the Board of Directors for the American Board of Obesity Medicine. Jens Walter reports consultancy and honoraria fees from Abbott Nutrition and PrecisionBiotics (Novonesis Group) and has received research funding from AgriFibre and Ingredion. He is a patent owner of *Limosilactobacillus reuteri* PB‐W1 (NCIMB 42835) and is a cofounder of Synbiotic Health. Ellen E. Blaak reports consultancy and honoraria fees from Abbott Nutrition and has received research funding from Next Food Collective, a public‐private partnership of Danone, DSM, and FrieslandCampina.

## Data Availability

Data sharing is not applicable to this article as no new data were created or analyzed in this study.
